# A Natural Organic Compound “Decursin” Has Both Antitumor and Renal Protective Effects: Treatment for Osteosarcoma

**DOI:** 10.1155/2023/5445802

**Published:** 2023-12-14

**Authors:** Daichi Hayashi, Toshiharu Shirai, Ryu Terauchi, Shinji Tsuchida, Naoki Mizoshiri, Yuki Mori, Seiji Shimomura, Osam Mazda, Kenji Takahashi

**Affiliations:** ^1^Department of Orthopaedics, Graduate School of Medical Science, Kyoto Prefectural University of Medicine, Kamigyo-ku, Kyoto 602-8566, Japan; ^2^Department of Immunology, Kyoto Prefectural University of Medicine, Kamigyo-ku, Kyoto 602-8566, Japan

## Abstract

Osteosarcoma is a rare malignant tumor that commonly occurs in children. Anticancer drugs, for example, cisplatin, aid in postsurgery recovery but induce side effects such as renal damage, affecting the life prognosis of patients. Decursin which is one of the bioactive components has been reported for its anti-inflammatory, antioxidant, and antitumor effects, but the effect on osteosarcoma is unexplained. In this study, the research theme was to examine the sensitizing effect of decursin and its influence on cisplatin-induced nephrotoxicity. The cell viability and half maximal inhibitory concentration (IC50), apoptosis induction, and effect on cell cycle and Akt pathways were examined. In vivo, we examine the effects of decursin on tumors and mice bodies. Additionally, the effects of the cisplatin-decursin combination were evaluated in vitro and in vivo. Decursin suppressed cell viability and induced apoptosis via the cell cycle. Decursin also inhibited the Akt pathway by suppressing the phosphorylation of Akt. It enhanced apoptosis induction and lowered cell viability in combination with cisplatin. The increasing tumor volume was suppressed in the decursin-administrated group with further suppression in combination with cisplatin compared to sole cisplatin administration. The decrease in renal function and renal epithelial cell damage caused by cisplatin was improved by the combinatorial treatment with decursin. Therefore, decursin demonstrated an antitumor effect on the osteosarcoma cells and a renal protective effect in combination with cisplatin. Therefore, decursin is a prospective therapeutic agent against osteosarcoma.

## 1. Introduction

Osteosarcoma accounts for 0.2% of all malignant tumors and is called rare cancer. The frequency of occurrence is about 2 per million people per year [1]. Osteosarcoma also accounts for about 5% of malignancies in children and is most prevalent in the age group of 10–20 years. Until the 1970s, limb amputation was the only treatment and the 5-year survival rate was 10–20%. In the 1980s, multiple anticancer drugs such as doxorubicin and cisplatin started being utilized, and chemotherapy using these drugs improved the 5-year survival rate to 60–70% [2]. Further advances in therapy made it possible to preserve the affected limb via tumor shrinkage. Multidisciplinary treatment, combining chemotherapy and surgery, is now the standard treatment. However, new anticancer drugs for osteosarcoma have never appeared in the last four decades mainly due to the significantly lesser number of cases of osteosarcoma compared to other cancers and the requirement of an excessive amount of time, expenditure, and labor [3, 4].

The problem with existing chemotherapy in the treatment of osteosarcoma is that the administration of large doses over a long period of time causes serious side effects such as myocardial damage, renal damage, and bone marrow suppression [5]. If a serious side effect occurs, a sufficient amount of anticancer drug cannot be administered, and treatment must be discontinued. As a result, sarcoma progression causes a decrease in survival time. In addition, dialysis treatment associated with renal damage and peripheral neuropathy often decreases the quality of life [6].

Cisplatin is an anticancer drug used in chemotherapy for many malignant tumors and is one of the standard treatments of choice for osteosarcoma [4]. Cisplatin, when taken up by the cancer cell, inhibits replication by binding to the double-stranded DNA cross-linking and leads the cancer cell to apoptosis arresting the cell cycle in the G2 phase [7]. However, high doses of cisplatin produce reactive oxygen species that damage the renal tubules [8]. Therefore, countermeasures against the side effects of cisplatin are required [9, 10].

Recently, anticancer agents derived from natural organic compounds have been utilized, such as trabectedin and eribulin for soft-tissue sarcomas [11, 12]. *Angelica gigas* Nakai (AGN) is a natural plant that grows in Southeast Asia and is used as a Chinese herbal medicine [13, 14]. It is effective against dysmenorrhea, amenorrhea, and menopausal syndrome [13]. Decursin, which is mostly extracted from AGN, has antioxidant, antibacterial, anti-inflammatory, and amnestic properties [15–18]. The antioxidant activity of AGN imposes a palliative effect on drug-induced neurotoxicity and renal dysfunction [19]. Furthermore, it has been known to have antitumor effects in various cancers [20–23]. However, no one has reported the effect of decursin in osteosarcoma.

The purpose of this study was to evaluate the effect of decursin on osteosarcoma and to investigate the sensitizing effect and relationship with renal damage of decursin in combination with cisplatin. Ultimately, aiming for clinical application, the goal is to improve the 5-year survival rate, which has remained flat, and to help patients who cannot receive adequate doses of anticancer drugs due to treatment resistance or side effects. In this study, only the relationship with cisplatin was evaluated, and the relationship with other drugs used concomitantly in clinical practice is unknown.

## 2. Materials and Methods

### 2.1. Cell Lines

The human osteosarcoma cells (143B and MG63: catalog nos. CRL1427 and 8303) and human osteoblasts (NHOst: catalog no. CRL11372) were purchased from the American Type Culture Collection (Rockville, MD, USA). As a medium, Dulbecco's Modified Eagle's Medium (Nacalai Tesque, Kyoto, Japan) containing 10% fetal bovine serum (Equitech-Bio, Kerrville, TX, USA) and penicillin and streptomycin (Nacalai Tesque) were used.

### 2.2. Reagents

We purchased decursin (catalog no. SML0786-5MG) from Sigma (St. Louis, MO, USA) and cisplatin (catalog no. 033-20091) from Wako Co., Ltd., Japan.

### 2.3. Antibodies

We purchased Akt and p-Akt (catalog nos. 4691S and 9271T), Bax and Bcl-2 (2772T and 2872T), mTOR and p-mTOR (2983S and 5536S), nuclear factor *κ*B (NF-kB) p65 and 0-NF-kB (8242S and 3033S), Cox2 (12282S), 4EBP1 and p-4EBP1 (#9644 and #9451S), Cyclin D1 (2978S), CDK6 (13331T), and *β*-actin (A2228) as a primary from Cell Signaling Technology (Danvers, MA, USA) and anti-mouse and rabbit IgG (catalog no. A4416 and A0545) as a secondary antibody from Sigma Aldrich.

### 2.4. Cell Viability Assay

Cells were seeded and incubated for 24 hours. MT cell viability substrate, Nanoluc® enzyme (RealTime-Glo MT Cell Viability Assay Kit: catalog no. G9713 from Promega, Madison, WI, USA), and various concentrations of decursin were administered. After 12, 24, and 48 hours, luminescence was measured. To evaluate the effectiveness of the biological inhibitory effect of decursin, the 50% inhibition concentration (IC50) was calculated with the obtained experimental data. In addition, the cell viability of decursin and cisplatin at various concentrations and times was measured with 143B and MG63, respectively. The drug interaction between these two drugs was examined. The evaluation method was calculated by loading the obtained data into dedicated computer software “CompuSyn.”

### 2.5. Annexin V/PI Staining Assay

Cells were seeded and decursin was administered after 24 hours. 24 and 48 hours later, Annexin V-FITC (1 *μ*l) and PI solution (0.5 *μ*l) which was purchased from Nacalai Tesque (catalog no. 15342-54) were stained for 20 min. Following incubation, the cell status was analyzed by flow cytometry. 10000 cells were recorded for each sample.

### 2.6. Caspase Activity

Caspase-3 activity was measured 24 and 48 hours after administration of decursin with the Caspase-Glo 3/7 Assay (catalog no. G8090 from Promega).

### 2.7. Cell Cycle Assay

*Cell Cycle Assay Solution Deep Red*. After administration of decursin, we stained the cells with 2 *μ*l cell cycle assay solution deep red (catalog no. C548 from Dojindo). Following incubation, the cell status was analyzed. For analysis, 10000 cells were recorded.

*EdU Assay*. After administration of decursin, cells were exposed to 20 *μ*M EdU (catalog no. C10337 from Thermo Fisher Scientific). The cells were analyzed 10000 counts per sample by flow cytometry.

### 2.8. Western Blot Analysis

We seeded cells and harvested following treatment with decursin for 24 and 48 hours. After removing the culture medium, RIPA buffer kit, SDS solution (catalog no. 08714-04 from Nacalai Tesque), and solubilization were administered (each well with 150 *μ*L) and centrifuged. Using BCA assay, proteins were loaded onto polyacrylamide gels for SDS PAGE. Protein samples were separated on 10% Bis-Tris Gel NuPAGE® electrophoresis (catalog no. NP0302BOX from Thermo Fisher Scientific). After dry-blotting, the membranes were soaked to the Blocking One solution (catalog no. 03953-95, Nacalai Tesque) for 60–90 min and to the solution containing the following primary antibodies (1 : 4000 dilutions each), including *β*-actin as a control for more than 8 h. Membranes were washed and reacted for 60 min with secondary antibodies (1 : 4000 dilutions each). Using Chemi Lumi One imager (catalog no. 07880-70 from Nacalai Tesque), protein bands were visualized and detected.

### 2.9. Animals

According to the NIH Guide for the Care and Use of Laboratory Animals, BALB/C-nu/nu mice (4 week old, females) from Shimizu Laboratory Supplies (Kyoto, Japan) were bred in the Experimental Animals Committee, Kyoto Prefectural University of Medicine (code nos. M2019-523 and M2020-289).

#### 2.9.1. Tumor Growth Assay In Vivo

After 1 week of breeding, 2.0 × 10^6^ cells/100 *μ*L were administrated into the subcutaneous area hypodermis of the back in each mouse. From the day when the tumor size reached an average of 50 mm^3^, we intraperitoneally (i.p.) administrated mice with decursin (10 mg/kg) and cisplatin (2 or 4 mg/kg), every 3 days for 21 days. As a control group (each group contained 5 mice), mice were administrated a solution of 10% DMSO and 90% PBS i.p. Tumor size was measured on the day of drug administration. All mice underwent arterial blood tests under inhalation anesthesia on day 21 and then sacrificed. After that, the subcutaneous tumor and renal samples were removed.

#### 2.9.2. Side Effects

We performed blood tests in mice to examine the liver and renal function. For pathological evaluation in the kidney, tumor and renal specimens stained with hematoxylin-eosin (HE) were made at Sept Sapie (Tokyo, Japan).

### 2.10. Statistical Analysis

Each experiment was repeated 3 times or more. Each sample was represented as means ± SD. To examine statistical differences among the groups, ANOVA was used. In all analyses, we considered that *P* < 0.05 (^*∗*^*P* < 0.05, ^*∗∗*^*P* < 0.01) was statistically significant.

## 3. Results

### 3.1. Decursin Suppresses Cell Proliferation in Human Osteosarcoma Cells

**Figure 1:**
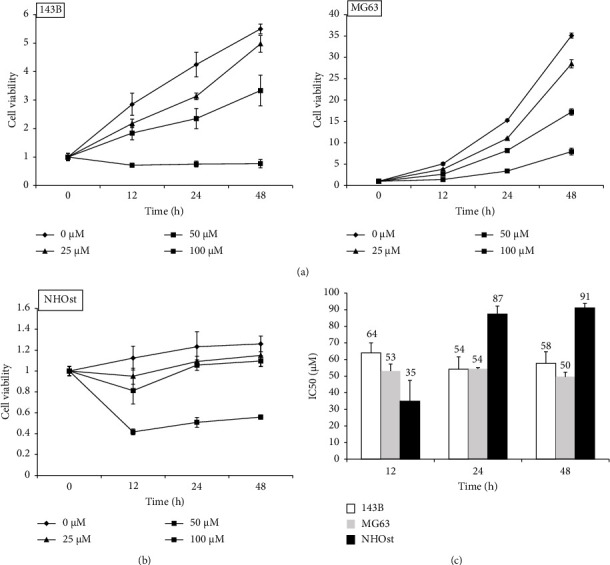
Effect of decursin on the viability of osteosarcoma and osteoblasts. (a) Human osteosarcoma cells (143B and MG63) were treated with various doses (0–100 *µ*M) of decursin for 12, 24, and 48 h. Cell viability was assessed using the RealTime-Glo MT cell viability assay (*n* = 4). (b) Human osteoblast cells (NHOst) were treated with various doses (0–100 *µ*M) of decursin for 12, 24, and 48 h (*n* = 4). (c) IC50 was calculated from the cell viability data.

After decursin administration (0, 25, 50, and 100 *μ*M), we measured the cell viability at 12, 24, and 48 h. In both 143B and MG63, decursin reduced cell viability in a concentration-dependent manner (Figure 1(a)). In normal osteoblasts, a decrease of the cell viability was observed only at 100 *μ*M and cell viability at other concentrations was comparable to that of the control (Figure 1(b)). IC50 values in each cell were as follows: 143B, 54.2 and 57.7 *μ*M (24 and 48 h); MG63, 54.3 and 49.7 *μ*M; and NHOst, 87.3 and 91.1 *μ*M (Figure 1(c)).

### 3.2. Decursin Has the Ability to Induce Apoptosis in Human Osteosarcoma Cells

**Figure 2:**
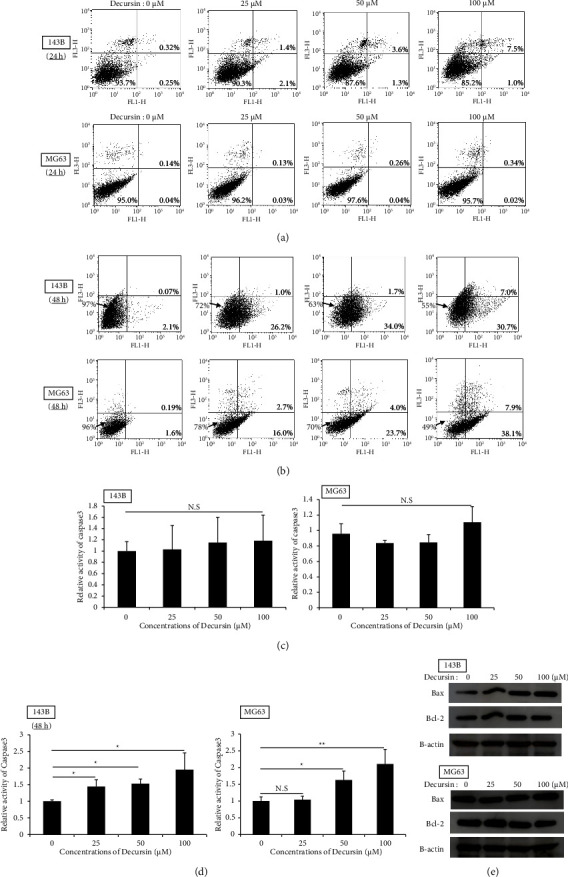
Decursin induces apoptosis in osteosarcoma cells. (a, b) Human osteosarcoma cells (143B and MG63) were treated with various doses (0–100 *µ*M) of decursin for 24 and 48 h. Apoptosis was assessed via Annexin V-FITC/propidium iodide double staining using flow cytometry. (c, d) Caspase-3 activity was measured using a Caspase-Glo assay kit. The relative ratios are presented. Caspase-3 activity was measured 24 and 48 h after administration of decursin. (e) Western blot analysis was performed using antibodies against Bcl-2 and Bax. *β*-Actin was used as the loading control. In (a–d), data are expressed as mean ± standard deviation (*n* = 4). ^*∗*^*P* < 0.05 and ^*∗∗*^*P* < 0.01, as control. N.S.: not significant.

We analyzed cell changes after decursin administration by flow cytometry at 24 and 48 h. At 24 h, decursin increased apoptosis cells in early and late stages compared to the control in 143B (Control: 0.57%, 25 *µ*M: 3.5%, 50 *µ*M: 4.9%, and 100 *µ*M: 8.5%) (Figure 2(a)). In MG63, there was no change due to concentration (Control: 0.18%, 25 *µ*M: 0.16%, 50 *µ*M: 0.3%, and 100 *µ*M: 0.36%). At 48 h, both 143B and MG63 increased in a concentration-dependent manner (143B control: 2.8%, 25 *μ*M: 27.2%, 50 *μ*M: 35.7%, and 100 *μ*M: 37.7%; MG63 control: 1.8%, 25 *μ*M: 18.7%, 50 *μ*M: 27.7%, and 100 *μ*M: 46.0%) (Figure 2(b)). In evaluation of caspase-3 located downstream of the apoptotic pathway, there was no clear difference between 143B and MG63 in number of apoptotic cells compared to the control at 24 h (Figure 2(c)). At 48 h, caspase-3 activation was observed in both 143B and MG63 in a concentration-dependent manner (Figure 2(d)). Furthermore, using western blot analysis, we evaluated Bax and Bcl-2 as apoptosis-related proteins. At 24 h, decursin increased the expression of the apoptosis-promoting factor (Bax) and did not increase that of the apoptotic inhibitor (Bcl-2). As a result, that of Bax/Bcl-2 ratio increased (Figure 2(e)).

### 3.3. Decursin Acts on the G0/G1 Phase

**Figure 3:**
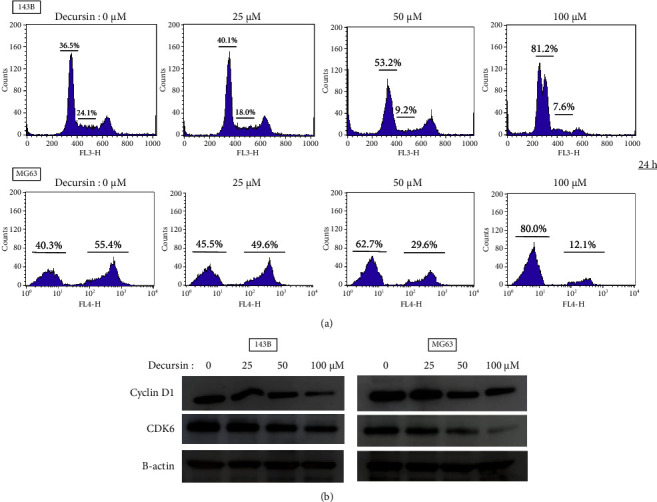
Decursin influences the cell cycle in osteosarcoma cells. (a) The human osteosarcoma cells (143B and MG63) were treated with various doses (0–100 *µ*M) of decursin for 24 h. The percentage of S period cells was calculated by the cell cycle assay solution deep red and EdU assay using flow cytometry. (b) Western blot analysis was performed using antibodies against cyclin D1, and CDK6 was used as the loading control. In (a), data are expressed as mean ± standard deviation (*n* = 4).

The cell cycle was evaluated by deep red cell cycle assay for 143B and EdU assay for Mg63. In both osteosarcoma cells, the number of G0/G1 phase cells was increased by decursin administration (143B control: 36.5%, 25 *μ*M: 40.1%, 50 *μ*M: 53.2%, and 100 *μ*M: 81.2%; MG63 control: 40.3%, 25 *μ*M: 45.5%, 50 *μ*M: 62.7%, and 100 *μ*M: 80%) and decreased in the S-phase (143B control: 24.1%, 25 *μ*M: 18.0%, 50 *μ*M: 9.2%, and 100 *μ*M: 7.6%; MG63 control: 55.4%, 25 *μ*M: 49.1%, 50 *μ*M: 29.6%, and 100 *μ*M: 12.1%). Evaluation of G2/M-phase was difficult (Figure 3(a)). In addition, we evaluated cyclin D1 and CDK6 proteins which were loaded downstream of the G0/G1 phase and involved in cell proliferation. Decursin reduced the expression of cyclin D1 and CDK6 (Figure 3(b)).

### 3.4. Decursin Acts on the Signaling Pathways Involved in Cell Proliferation

**Figure 4:**
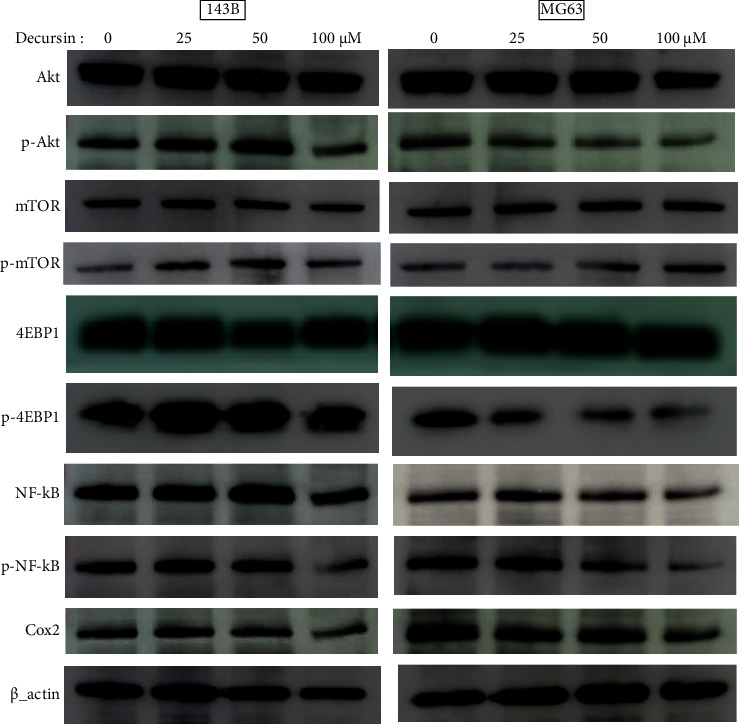
Signal pathway of decursin. Western blot analysis was performed using antibodies against phospho-Akt, total Akt, phospho-NF-*κ*B, total NF-*κ*B, phospho-mTOR, total mTOR, phospho-4EBP1, total 4EBP1, and Cox2. *β*-Actin was used as the loading control.

We evaluated the Akt pathway (Akt, mTOR, NF-kB, 4EBP1, and Cox2) which is a pathway involved in cell proliferation. Decursin suppressed the phosphorylation of Akt and NF-*κ*B in 143B cells, as well as the expression of Cox2, at 100 *μ*M; no effect was observed in 143B cells at concentrations below 50 *μ*M (Figure 4). In MG63 cells, decursin suppressed the phosphorylated Akt and NF-*κ*B, as well as that of Cox2. mTOR and 4EBP1 located downstream of mTOR were not affected, in either cell line.

### 3.5. Decursin Exerts a Sensitizing Effect in Combination with Cisplatin

**Figure 5:**
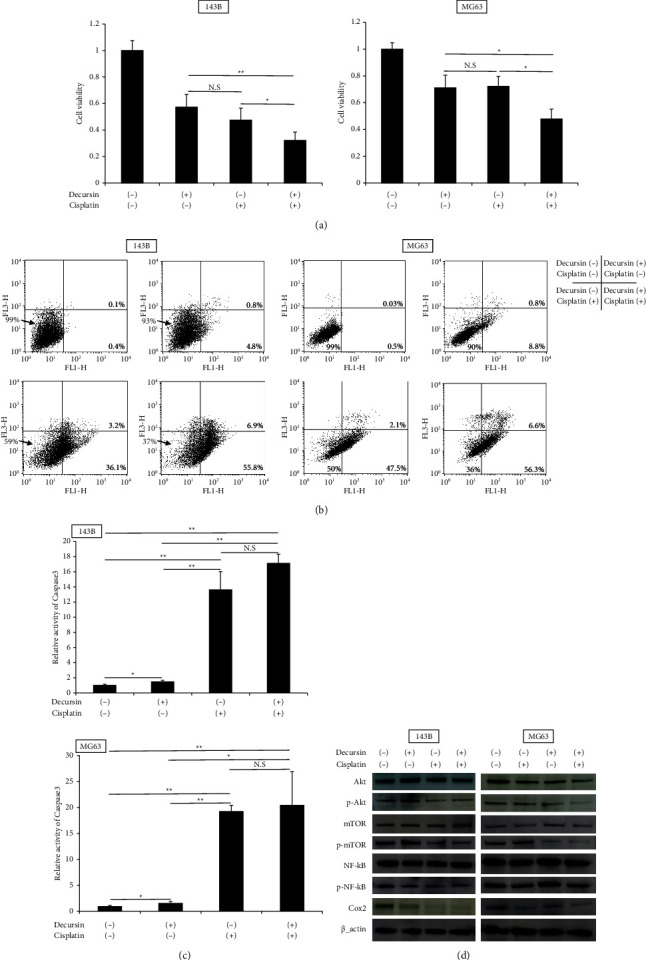
Synergy effects of decursin and cisplatin. (a) The 143B and MG63 cells were treated with 0 or 50 *μ*M of decursin and 0 or 5 *μ*M cisplatin for 48 h. The cell viability was assessed using the RealTime-Glo MT cell Viability assay (*n* = 4). (b) 143B and MG63 cells were administered decursin and/or cisplatin, after which flow cytometry was performed and the percentage of apoptotic cells was calculated at 48 h. Apoptosis was assessed via Annexin V-FITC/propidium iodide double staining, using flow cytometry. (c) Caspase-3 activity was measured using a Caspase-Glo assay kit. The relative ratios are presented. Caspase-3 activity was measured after 48 h of decursin and/or cisplatin treatment. (d) Western blot analysis was performed using various antibodies, including the same ones as those used in Figure 2, after decursin and/or cisplatin treatment. In (a, c), data are expressed as mean ± standard deviation (*n* = 4). ^*∗*^*P* < 0.05 and ^*∗∗*^*P* < 0.01. N.S.: not significant.

The combined effect of decursin (50 *μ*M) and cisplatin (5 *μ*M) was evaluated 48 h after administration of the two drugs in both the cell lines. In both 143B and MG63 cells, the combined use of decursin and cisplatin significantly suppressed cell viability compared to that of every individual agent (Figure 5(a)). When the combination index (CI), which is one of the evaluation indexes of the drug combination effect, was calculated, the CI of 143B cells was found to be 0.87 and that of MG63 was 0.84, at 48 h. The CI was <1 for both 143B and MG63 cells, and the effects of each drug were synergistic. In addition, cisplatin alone increased early apoptotic cells in the lower right of FACS. The combined use of the two agents further increased compared to cisplatin alone (143B control: 0.5%, cisplatin: 39.3%, and two drugs: 62.7%; MG63 control: 0.53%, cisplatin: 49.6%, and two drugs: 62.9%) (Figure 5(b)). In both 143B and MG63 cells, caspase-3 was significantly activated by cisplatin alone compared to the control. Even with decursin alone, it was comparable to the control. In the combined use of the two agents, a significant difference was observed from decursin, but not from cisplatin alone (Figure 5(c)). We evaluated the protein activity of the two agents using western blot analysis. Cisplatin alone reduced the protein expression related to Akt pathway, for example, Akt. -NF-*κ*B and Cox2. It also suppressed the phosphorylation of mTOR. In the combined use of the two agents, the expressions of p-NF-*κ*B and Cox2 in 143B were lower than those upon administration of decursin or cisplatin alone. The expression of p-Akt, p-NF-*κ*B, and Cox2 in MG63 was reduced compared to single-agent use (Figure 5(d)).

### 3.6. Decursin Exhibited a Sensitizing Effect In Vivo in Combination with Cisplatin

**Figure 6:**
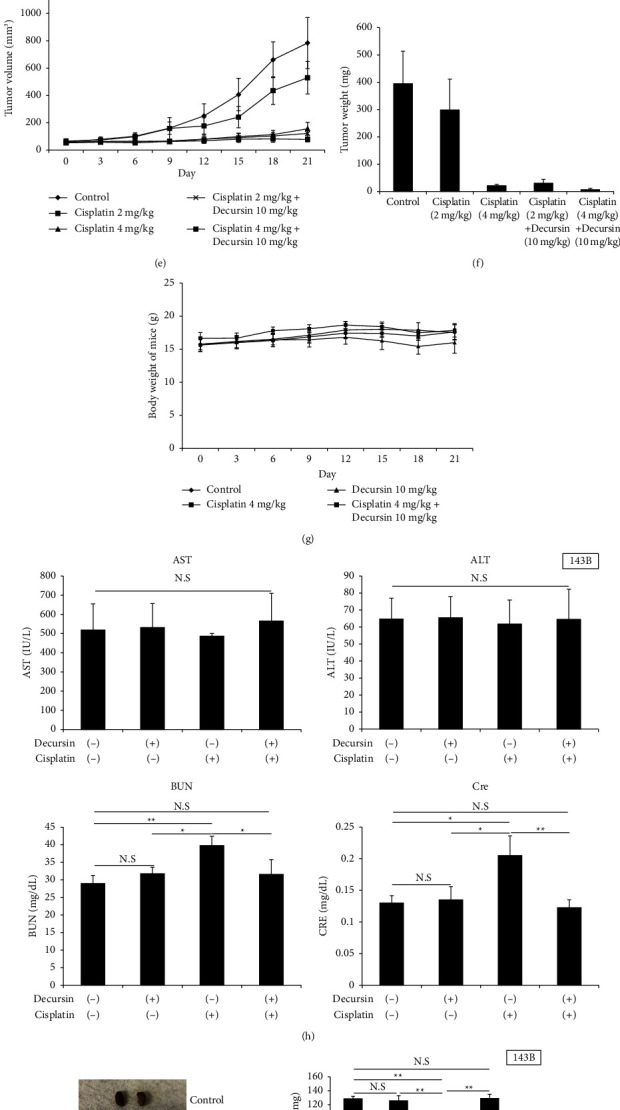
Effects of decursin and/or cisplatin in vivo. The synergistic effect, as well as the effect of adding cisplatin to decursin, was evaluated using xenografted mice. Decursin and cisplatin were administered intraperitoneally every three days after the tumor volume reached 50 mm³. (a) Tumor volumes of mice were measured after administration of decursin and cisplatin (*n* = 5). (b) The mice on day 21. The tumor volume was presented for every four out of five animals. (c, d) The tumor weights were measured on day 21. (e) The dose of cisplatin was increased, and the tumor volume was measured after administration of decursin and cisplatin (*n* = 5). (f) Under conditions of increased cisplatin, tumor weight was measured on day 21. (g) Body weight of mice was evaluated in the decursin and/or cisplatin (2 mg/kg or 4 mg/kg) group. (h) Blood samples (*n* = 5) were collected for assessment of liver and renal toxicity associated with decursin and/or cisplatin administration. ALT, alanine aminotransferase; AST, aspartate aminotransferase; BUN, blood urea nitrogen; Cre, creatinine. (i, j) Renal weight was measured in sacrificed rats (*n* = 5). (k, l) The resected kidney was stained with hematoxylin-eosin and observed at high or low magnification (scale bar = (k) 20 *μ*m; (l) 100 *μ*m). ^*∗*^*P* < 0.05 and ^*∗∗*^*P* < 0.01. N.S.: not significant.

The human osteosarcoma cells were subcutaneously transplanted in mice and effects of administration of decursin and cisplatin in combination were evaluated. Decursin and cisplatin were distributed at various concentrations, and we selected that which resulted in tumor volumes of 40–60% compared to controls at the final observation. The concentrations were cisplatin: 2 mg/kg and decursin: 10 mg/kg. As a result, four groups were set: control, cisplatin (2 mg/kg) single agent, decursin (10 mg/kg) single agent, and both decursin (10 mg/kg) and cisplatin (2 mg/kg) groups. No significant differences were observed in both the tumor volume and tumor weight at the final observation in the cisplatin single-agent group compared to the control group. The decursin single-agent group significantly suppressed tumor volume more than control group. Significant differences were observed in the two-agent group compared to the other three groups (Figures 6(a)–6(d)). Next, another five groups were set: control, cisplatin (2 or 4 mg/kg), and cisplatin (2 or 4 mg/kg) with decursin (10 mg/kg). Cisplatin suppressed the tumor volume growth in a concentration-dependent manner. By adding decursin to cisplatin (2 mg/kg), the same antitumor effect as cisplatin (4 mg/kg) was obtained. Moreover, the addition of decursin to cisplatin (4 mg/kg) significantly suppressed the increase in tumor volume compared to cisplatin (4 mg/kg) (Figures 6(e) and 6(f)). No clear difference in the changes in the body weight between the mice treated with high concentrations of cisplatin (4 mg/kg) and decursin and the two-agent groups was observed (Figure 6(g)).

### 3.7. Decursin Was Not Toxic to Living Organisms In Vivo

We collected blood tests from mouse arteries and evaluated the liver enzymes (AST and ALT) and renal function (BUN and creatinine). All values were within the normal range in the control group and decursin single-agent group. The values of BUN and creatinine were high with cisplatin administration alone and were within the normal range for the combination of the two drugs. AST levels were higher than normal in all the groups, including the controls. ALT levels were similar in all groups, and the values were within the normal range (Figure 6(h)).

### 3.8. Decursin Had a Protective Effect on Renal Tissue

We evaluated the renoprotective effect of decursin by creating the following groups: control, cisplatin (4 mg/kg), decursin (10 mg/kg), and a combination of the two drugs. Macroscopic findings of the excised renal tissue showed clear atrophy in the group receiving cisplatin alone, but no renal atrophy was observed with the combination of cisplatin and decursin (Figure 6(i)). Weight of the renal tissue was significantly reduced in the cisplatin single-agent group compared to control group. However, combined use with decursin improved renal tissue weight to the same level as control (Figure 6(j)). Pathological evaluation of the renal tissue using hematoxylin-eosin staining revealed atrophy in the cisplatin single-agent group with weak enlargement, but there was no renal tissue atrophy in the combination group (Figure 6(k)). In the strong enlargement, foam-like degeneration of the tubular epithelial cells in the proximal tubule was observed, only in the cisplatin single-agent group. In the cisplatin and decursin combinatorial group, the degenerative findings of the epithelial cells were similar to those in the normal tissue (Figure 6(l)).

## 4. Discussion

Decursin is among the natural organic compounds extracted from AGN. The results show that decursin has an antitumor effect on osteosarcoma and a sensitizing effect with cisplatin. Furthermore, it was revealed that decursin prevents cisplatin-induced renal damage.

There are some research reports that decursin has an anti-inflammatory effect on epidermal keratinocytes, an antifibrotic effect on the liver, and an antioxidant effect on renal epithelial cells [13, 17, 24]. In addition, antitumor effects have been reported in some cancers [25–27]. In this study, decursin suppressed the cell viability at 24–48 h in time- and concentration-dependent manner. However, it did not suppress the viability of the normal human osteoblast, NHOst. Also, the cause of decreased viability of the normal cells, 12 h after decursin administration, has not been investigated. The IC50 value of the malignant melanoma cell line (B16F10) was reported to be >100 *μ*M at 24 h and 60–80 *μ*M at 48 h after decursin administration. That of the prostate cancer cell line (DU145) was reported to be approximately 100 *μ*M at 24 h and 65–70 *μ*M at 48 h after decursin administration [28, 29]. The IC50 values of human osteosarcoma cells in this study were 54.2 *μ*M for 143B and 54.3 *μ*M for MG63, respectively, at 24 h and were 57.7 *μ*M and 49.7 *μ*M, respectively, at 48 h after decursin administration. The IC50 values of NHOst were 87.3 *μ*M at 24 h and 91.0 *μ*M at 48 h. The IC50 values of decursin for 143B and MG63 were lower than those of NHOst and other cancer cell lines. Therefore, we considered that decursin has a lower effect on the normal osteoblasts and is more effective in human osteosarcoma compared to the other cancer types.

Cell viability primarily reflects the effects of apoptosis and cell proliferation [30]. Decursin has been known to have an antitumor effect by inducing apoptosis in some cancers [20, 28, 31]. In this study, using Annexin V-PI staining, decursin did not appear to induce apoptosis in the human osteosarcoma cells at 24 h, but it induced apoptosis at 48 h in a concentration-dependent manner. Therefore, we consider that decursin induces apoptosis for more than 24 hours after administration against human osteosarcoma cells. The caspase pathway is one of the inducing apoptosis pathways. As a proapoptotic signal, caspases are divided into an initiator group (caspase-2, -8, and -9) and an effector group (caspase-3 and -7). It is known to affect cell membranes and induce apoptosis by activating effector caspases. In this study, we focused on caspase-3, an effector caspase, and evaluated the caspase activation. Decursin did not show clear activation of caspase-3, 24 h after administration, but the enzyme was activated in a concentration-dependent manner after 48 h. Therefore, we inferred that decursin induced apoptosis via the caspase pathway, among the apoptosis-inducing pathways. There were several proteins, for example, Bcl-2 and Bax, involved in caspase pathway [32]. Suppression of Bcl-2 is generally thought to increase Bax located downstream. Increased Bax/Bcl-2 ratio activates the caspase pathway and induces apoptosis [32]. The expression of Bcl-2 was constant regardless of the concentration of decursin after 24 h, but that of Bax increased in this study. As a result, the Bax/Bcl-2 ratio was increased. Therefore, we considered that Bcl-2 and Bax are upstream of the caspase pathway in apoptosis. In general, the cell cycle arrest is a prestage that induces apoptosis. Previous reports have reported that decursin acts on the cell cycle and is involved in antitumor effects in prostate cancer, bladder cancer, and colorectal cancer [29, 33]. In this study, decursin increased the number of cells in the G0/G1 phase and decreased in the S-phase on human osteosarcoma cell lines at 24 h. Decursin also acted on cyclin D1 and CDK6 that arrests the cell cycle in G0/G1 phase. We believe that decursin affects the cell cycle. Therefore, we considered that the reason for the decrease in cell viability was that the cells were in a dormant state, 24 h after the administration of decursin, which led to apoptosis after 48 h.

The PI3K/Akt pathway is known to be one of the pathways related to the growth of cancer cells. In addition, these pathways are intricately involved in cell proliferation and anticancer drug resistance [34, 35]. Decursin has an antitumor effect via NF-*κ*B in breast cancer and leukemia [22, 36]. It is known that phosphorylated Akt activates mTOR and NF-*κ*B, and mTOR is upstream of 4EBP1 and NF-kB is upstream of Cox2. mTOR integrates internal and external environmental information (such as growth factors and nutritional/energy status) and promotes cell proliferation and growth by protein synthesis and ribosome production. NF-*κ*B is a gene regulatory protein present in most animal cells and is involved in cell stress and inflammatory responses and cancer cell proliferation by apoptosis resistance [37–39]. Therefore, these proteins and their downstream factors such as 4EBP1 or Cox2 are regarded as important therapeutic target factors for cancer treatment. We consider that decursin has an antitumor effect by suppressing the expression of Akt pathway proteins such as p-Akt, p-NF-*κ*B, and Cox2 in this study.

Decursin has a sensitizing effect in combination with doxorubicin acting on caspase pathway in multiple myeloma [14]. In this study, administration of decursin and cisplatin that has the same effect as doxorubicin showed a clear sensitizing effect after 48 h. Cisplatin is known to induce apoptosis by arresting the G2 phase [40, 41]. In this study, the combined use of two agents increased the percentage of apoptotic cells compared to cisplatin. It is considered that the combined use of the two agents acted on both the G0/G1 and G2 phases to induce apoptosis sufficiently and enhance an antitumor effect. It is known that cisplatin acts on Akt pathway [42, 43]. In this study, administration of cisplatin suppressed the expression of p-Akt, p-mTOR, and p-NF-*κ*B as well as Cox2. When cisplatin and decursin were used in combination, the expression of p-NF-*κ*B and Cox2 was suppressed as compared to cisplatin or decursin alone. The Akt pathway is related to the expression of cyclin D1 and CDK6 in osteosarcoma [44]. In this study, decursin suppressed the expression of cyclin D1 and CDK6. Therefore, we considered that the mechanism of action on decursin may involve the Akt pathway to cyclin D1 and CDK6. However, we did not evaluate the experiment using inhibitors, and it was difficult to evaluate the interactions between the Akt pathway and the cyclin D1 to CDK6 pathway.

In vivo, according to Li et al., decursin was reported to suppress hepatocellular carcinoma growth by approximately 70% at a concentration of 30 mg/kg in BALB/c nude mice [26]. Kim et al. also reported that decursin, at the concentration of 10 mg/kg, suppressed the growth of malignant melanoma by approximately 50% in male C57BL/6J mice [28]. In this study, decursin suppressed the growth of osteosarcoma by approximately 75% in 143B at a same concentration. Therefore, we consider that decursin has an antitumor effect in osteosarcoma which is greater than that in other cancer types in vivo.

Cisplatin has a concentration-dependent antitumor effect, but it has serious side effects depending on the concentration of dose administered [45]. In this study, even if the dose of cisplatin was halved from 4 to 2 mg/kg, the combined use of decursin showed the same antitumor effect as cisplatin (4 mg/kg) alone. Therefore, decursin is a drug that makes it possible to reduce the dose of cisplatin. As a result, it can be expected that decursin has the ability to reduce the cisplatin-induced side effects.

The effect of a drug on a living body may be evaluated by changes in dietary intake and changes in body weight [46]. We verified the weight change on the living body, but the weight of mice at the final observation was not significantly different in all groups. Furthermore, the concentrations of AST, ALT, creatinine, and BUN were measured via blood tests, but no effect on the liver or renal function was observed with administration of decursin alone. Therefore, decursin was considered to be a drug that exerts an antitumor effect at an appropriate concentration and is less likely to cause side effects to the body.

Cisplatin is a clinically important therapeutic drug in cancer treatment, but renal damage is known to be a serious side effect [47]. Cisplatin has been reported to be taken up by the renal epithelial cells via the organic cation transporter (OCT) and to act on the release of reactive oxygen species and Ca^2+^ concentration to cause toxicity [48]. In this study, administration of cisplatin (4 mg/kg) showed a sufficient tumor growth inhibitory effect on the human osteosarcoma cells, but blood tests showed a decrease in renal function, and histological evaluation showed renal atrophy and degeneration of proximal tubule cells. This result is consistent with the previously reported renal damage associated with cisplatin administration. Decursin has also been reported to prevent drug-induced renal epithelial cell damage in vitro, owing to its antioxidant activity [15, 24]. However, there are no reports that decursin prevents cisplatin-induced nephropathy in vivo. In this study, we certified the effect of decursin in combination with cisplatin in vivo to prevent renal atrophy and renal dysfunction for the first time. Decursin has a sensitizing effect with cisplatin, as well as a nephroprotective effect, in vivo; therefore, it has potential as a new therapeutic agent for osteosarcoma.

## 5. Conclusion and Future Perspective

This is the first report to investigate the antitumor effect of decursin and the sensitizing effect and side effects with cisplatin on osteosarcoma. Decursin exerted antitumor effect by acting on the cell cycle via the Akt pathway and inducing apoptosis. Since decursin has little effect on normal cells and tissues and has reduced the side effects of cisplatin, we believe that it will bring beneficial results when used in combination with current chemotherapy. In the future, we plan to evaluate the relationship with other anticancer drugs such as doxorubicin and methotrexate.

## Data Availability

The research data including figure data used to support the findings of this study are available from the corresponding author upon request.
